# Advances in the diagnosis and treatment of urethral metastasis after urothelial carcinoma surgery: A case series and literature review of 3 total ureterectomy cases

**DOI:** 10.1097/MD.0000000000042329

**Published:** 2025-05-16

**Authors:** Jiajun Chen, Weihao Wang, Mengyao Li, Shouhua Pan, Keyuan Zhao

**Affiliations:** a Department of Urology, Shaoxing People’s Hospital, Shaoxing, Zhejiang, China; b Department of Pathology, Shaoxing People’s Hospital, Shaoxing, China

**Keywords:** GC chemotherapy regimen, PD-1 inhibitors+ADC combination therapy, total urethrectomy, urethral metastasis, urothelial carcinoma

## Abstract

**Rationale:**

Urethral metastasis recurrence after surgery for urothelial carcinoma (UC) is a common clinical challenge, yet there is no established optimal treatment strategy to effectively control postoperative metastasis and recurrence. This case report evaluates the efficacy of total urethrectomy combined with adjuvant chemotherapy and PD-1 + antibody–drug conjugate (ADC) immunotherapy in managing UC urethral metastasis.

**Patient concerns:**

Three patients with a history of UC surgery presented with urethral metastasis recurrence, raising concerns about disease progression, treatment options, and long-term survival.

**Diagnoses:**

All 3 patients were definitively diagnosed with UC urethral metastasis recurrence based on clinical, imaging, and histopathological examinations.

**Interventions:**

Each patient underwent total urethrectomy followed by adjuvant chemotherapy and PD-1 inhibitor + ADC combination immunotherapy.

**Outcomes:**

Post-treatment follow-up, including serum tumor marker assessments and imaging studies, showed no significant recurrence. The patients exhibited prolonged overall survival and maintained a satisfactory postoperative quality of life.

**Lessons:**

Total urethrectomy combined with chemotherapy and PD-1+ADC immunotherapy may significantly improve outcomes in UC patients with postoperative urethral metastasis recurrence. This multimodal approach warrants further investigation in larger clinical studies.

## 1. Introduction

Urothelial carcinoma (UC) accounts for over 90% of bladder cancer cases, and the standard treatment for UC is radical nephroureterectomy combined with bladder cuff excision and lymph node dissection. However, more than 50% of UC cases will experience urethral metastasis recurrence after surgery.^[1]^ Most recurrent cases are due to insufficient tumor resection, but there are also cases where tumor cells spread distally during surgery.^[2]^ Due to limited literature reporting, the systemic treatment of patients with urethral metastasis recurrence after surgery for UC poses a significant challenge.^[3]^ In response to this phenomenon, understanding the mechanisms of urethral metastasis after UC surgery and effective diagnostic and systemic treatment strategies are crucial for improving patient prognosis and guiding clinical management. This article reports 3 cases of postoperative urethral metastasis in patients with urinary tract epithelial cell carcinoma, presenting as urethral polyps, urethral pain, and penile pain, respectively. Serum tumor markers, including cancer-associated antigen 19-9, carcinoembryonic antigen, alpha-fetoprotein, and beta-human chorionic gonadotropin, showed no elevation. One female patient had local recurrence 1 year after urethral lesion excision surgery for a bleeding urethral mass, leading to subsequent urethrectomy and partial vaginal excision. Two male patients underwent total penectomy. Postoperative pathology results indicated invasive metastasis of urinary tract epithelial carcinoma. We reviewed the medical records of these 3 cases and discussed the diagnosis and treatment modalities for urethral metastasis after surgery for UC.

## 2. Case presentation

### 2.1. Case 1

**Figure 1.**
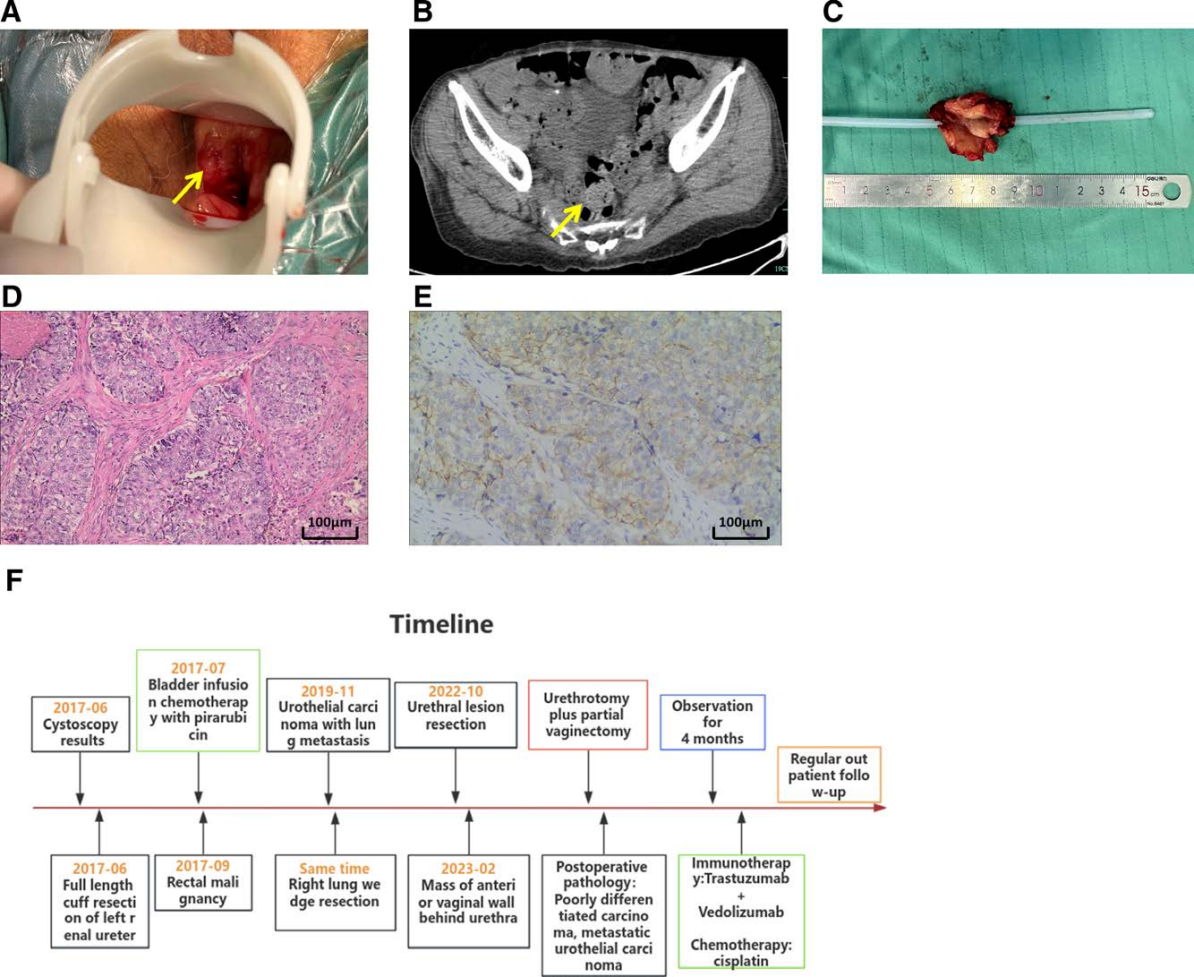
(A) Introital examination reveals a firm mass behind the urethra on the anterior wall of the vagina, characterized by a hardened texture, with surface skin fissures oozing blood. (B) Contrast-enhanced CT scan of the entire abdomen reveals the presence of a soft tissue mass in the perineal region. (C) Surgical image of urethrectomy, depicting a mass with a diameter of 4 cm. (D) Histopathological section, the tumor cells exhibit a papillary and clump-like distribution, with focal areas showing necrosis. There is significant cellular pleomorphism, and the cell nuclei are slightly vacuolated with prominent nucleoli. Mitotic figures are observed (HE staining, 100×). (E) The immunohistochemical staining results indicate CerbB-2 expression at 2+. (F) The timeline of information reported in case 1. CT = computed tomography, HE = hematoxylin-eosin.

In February 2023, an 82-year-old female patient was admitted due to a urethral caruncle after surgery in April and vaginal bleeding for 3 days. She had a history of malignant rectal tumor. Previously, she was diagnosed with a malignant renal pelvic tumor and underwent left renal ureteral full-length bladder cuff-like excision surgery in June 2017. The postoperative pathology revealed high-grade renal pelvic UC (tumor size 1.2 × 1.5 cm), with the tumor restaged as T1N1M0, and intravesical instillation of pirarubicin was performed. In 2019, she underwent right middle lobe resection for lung cancer in another hospital. Postoperative pathology suggested poorly differentiated carcinoma consistent with high-grade UC metastasis, without subsequent treatment. In October 2022, she underwent urethral lesion excision surgery at Shaoxing People’s Hospital, without adjuvant therapy postoperatively. On examination with a speculum, a hard mass was observed in the posterior urethra and anterior vaginal wall, with cracked bleeding on the surface (Fig. 1A). Routine laboratory tests, liver and kidney function, and tumor markers including cancer-associated antigen 19-9, carcinoembryonic antigen, alpha-fetoprotein, beta-human chorionic gonadotropin, and squamous cell carcinoma antigen showed no significant abnormalities, with slightly elevated squamous cell carcinoma. Abdominal contrast-enhanced computed tomography (CT) revealed a soft tissue mass in the perineum, measuring 19 × 31 mm, showing significant enhancement with unclear borders (Fig. 1B), indicating tumor recurrence and metastasis. After comprehensive multidisciplinary team discussion and patient consent, it was decided to perform a repeat urethrectomy with partial vaginal excision. The gross specimen postoperatively showed no obvious capsule, with a diameter of approximately 4 cm papillary-like mass (Fig. 1C). Pathological results indicated poor cellular differentiation, suggesting poorly differentiated carcinoma, consistent with UC metastasis. The tumor cells exhibit papillary and nodular distribution, with focal areas of necrosis. There is significant cellular pleomorphism, with nuclei showing slight vacuolization and prominent nucleoli. Mitotic figures are visible (Fig. 1D). Additionally, the immunohistochemistry staining results reveal CerbB-2 (v‐erb‐b2 erythroblastic leukemia viral oncogene homolog 2, neuro/glioblastoma-derived oncogene homolog [avian]) expression as 2 + in the excised tissue sample (Fig. 1E). Four months postoperatively, the patient received immunotherapy with toripalimab at a dose of 240 mg IV and disitamab vedotin for injection at a dose of 120 mg IV, along with chemotherapy using cisplatin at a dose of 30 mg IV. The patient’s most recent follow-up on February 10, 2024, showed no recurrence of vaginal bleeding symptoms, and magnetic resonance imaging examination revealed no local or distant recurrence. Additionally, the Eastern Cooperative Oncology Group (ECOG) Performance Status Scale^[4]^ was used to assess the patient’s postoperative quality of life, with a score of 1, indicating satisfactory postoperative quality of life. Currently, the patient undergoes regular immunotherapy combined with chemotherapy in our department. The timeline of the patient’s disease treatment is detailed in Figure 1F.

### 2.2. Case 2

**Figure 2.**
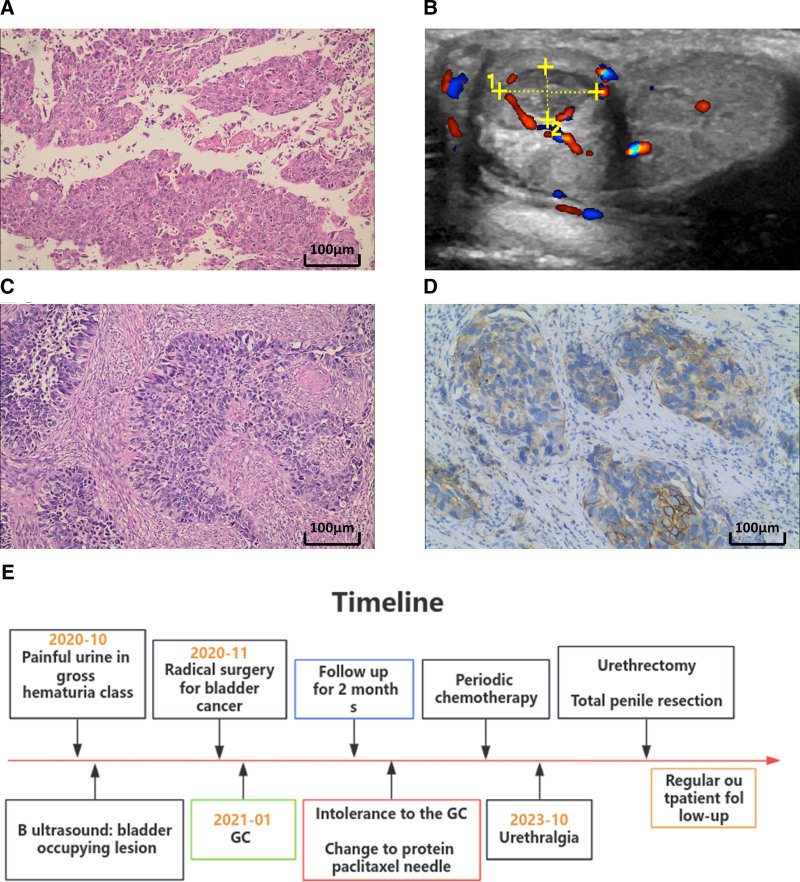
(A) Bladder tissue histopathology section, the tumor cells display a lamellar distribution with poor cell adhesion. There is marked cellular pleomorphism, and the cell nuclei are large, deeply stained, with evident nucleoli (HE staining, 100×). (B) Superficial ultrasound examination of the penis reveals a mass within the corpora cavernosa. (C) Penile tissue histopathological section, the tumor cells exhibit a lamellar distribution, accompanied by loss of cell polarity. The cell nuclei are large, deeply stained, and show significant pleomorphism, with observable mitotic figures (HE staining, 100×). (D) The immunohistochemical staining results indicate CerbB-2 expression at 2+. (E) The timeline of information reported in case 2. HE = hematoxylin-eosin.

In October 2023, a 70-year-old male presented with urethral pain lasting more than a month. In 2020, he was diagnosed with bladder malignancy and underwent laparoscopic radical cystectomy at our hospital. Postoperative pathology revealed low-grade UC (size 3.5 × 3.0 × 0.6 cm). The tumor cells exhibited a leaf-like distribution with poor cellular adhesion, demonstrating significant cellular pleomorphism (Fig. 2A), with tumor staging classified as T3aN1M0. Two months postoperatively, he received the gemcitabine and cisplatin (GC) regimen. Around 4 months later, due to intolerance, the chemotherapy was switched to paclitaxel injection (300 mg/q3w) monthly. Physical examination showed a Brick bladder stoma on the right abdomen, with pale yellow urine flow and obvious tenderness of the penile corpus spongiosum. Laboratory tests were normal, and penile ultrasound revealed a well-defined hypoechoic area (85 mm) with minimal blood flow (Fig. 2B). With patient consent, he underwent urethrectomy and complete penile resection. Under the microscope, tumor cells appeared to have a sheet-like distribution with loss of cellular polarity. The cell nuclei were large, deeply stained, and exhibited significant pleomorphism, with visible mitotic figures. Pathology confirmed recurrent tumor infiltrating the penile corpus spongiosum (4 × 2 cm) with negative margins (Fig. 2C). The immunohistochemistry staining results indicated CerbB-2 expression as 2 + in the excised tissue sample (Fig. 2D). The patient recovered without complications, and as of February 11, 2024, reported improved urethral pain, with an ECOG score of 1, indicating good postoperative quality of life. The timeline of the patient’s disease treatment is detailed in Figure 2E.

### 2.3. Case 3

**Figure 3.**
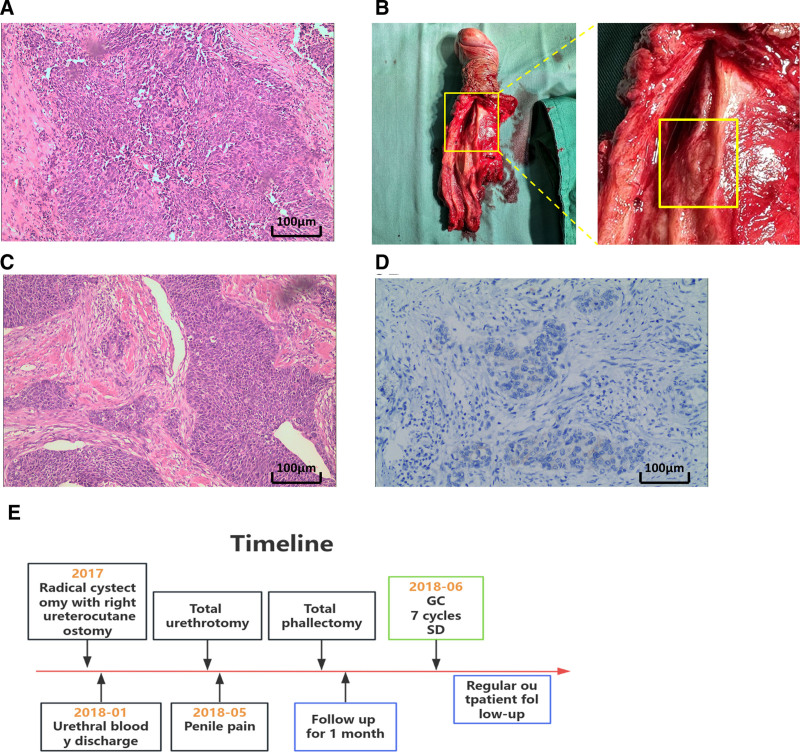
(A) Urethral tissue histopathological section, the tumor cell clusters with fibrovascular papillary axis demonstrate a lamellar fusion, accompanied by loss of cell polarity, irregular morphology, and prominent nucleoli (HE staining, 100×). (B) Surgical image of complete penectomy, showing tumorous lesions within the corpora cavernosa of the penis. (C) Penile tissue histopathological section, the tumor cells are relatively orderly arranged, with partial loss of polarity and somewhat irregular cellular morphology (HE staining, 100×). (D) The immunohistochemistry staining results indicate negative expression of CerbB-2. (E) The timeline of information reported in case 3. HE = hematoxylin-eosin.

A 70-year-old male patient was admitted in May 2018 reporting pain and discomfort in the penis that had lasted for 1 month. Previously, the patient was diagnosed with left ureteral infiltrating UC and underwent complete cystotomy and right ureteral dermostomy in our hospital without postoperative adjuvant treatment. In January 2018, the patient underwent a total urethral resection in our hospital due to hemorrhagic urethral secretions, and the postoperative pathological diagnosis was invasive UC. Under the microscope, tumor cell clusters containing fibrovascular cores appeared to merge into sheet-like formations (Fig. 3A). Physical examination revealed tenderness of the cavernous body of the urethra, and knock pain in the bilateral kidney area was negative. After discussion in the urology group and with the consent of the patient, a complete penile amputation was performed. During the operation, complete gross specimens of the penis and tumor-like lesions of the cavernous body of the penis were observed (Fig. 3B). Pathological examination revealed infiltration and metastasis of penile UC, with vascular cancer thrombus (diameter 2.5 × 2 × 1.5 cm) visible. Under the microscope, tumor cells were relatively orderly arranged, with some loss of polarity, and exhibited somewhat irregular cell morphology (Fig. 3C). Furthermore, the immunohistochemistry staining results suggested negative expression of CerbB-2 (Fig. 3D). One month after surgery, chemotherapy was administered using the GC regimen consisting of GC once every other month, totaling 7 sessions. At present, the patient regularly visits the outpatient clinic, with the last visit on February 9, 2024, showing no local recurrence and metastasis of the tumor, and the ECOG score was 0. The timeline of the patient’s disease treatment is detailed in Figure 3E.

## 3. Discussion

The standard treatment for urinary tract epithelial carcinoma involves radical nephroureterectomy combined with bladder cuff excision and lymph node dissection. Postoperatively, approximately 50% of patients with urinary tract epithelial carcinoma experience urethral metastatic recurrence, with the majority occurring within 5 years after surgery, and the incidence rate is approximately 25%.^[5]^ The 3 patients reported metastasis and recurrence within 5 years. The reasons for metastasis and recurrence were the large size of the primary tumor, the insufficient resection scope, the lack of postoperative pathological examination of the resection margin, the lack of adjuvant radiotherapy and chemotherapy in the tumor bed area, and the lack of regular postoperative follow-up. In addition, some scholars have hypothesized that in the presence of upper urinary tract tumors, tumor cells are implanted from the upper urinary tract to the distal urethra, which may also be caused by intraoperative cancer cell extravasation or the spread of undetected upper urinary tract epithelial cells.^[6]^

Several auxiliary examinations are crucial for UC diagnosis. CT urography provides higher sensitivity for diagnosing UC and can assess lymph node invasion and distant metastasis, making it the most accurate imaging method for diagnosing UC.^[7]^ Case 1 was diagnosed with UC urethral metastasis through abdominal CT. Ureteroscopy uses the Karl Storz endoscopic system to enhance the contrast between mucosal capillaries and vessels, tissues, and structures, providing clearer graphics for detecting pathological areas.^[8]^ However, it requires anesthesia and carries the risk of ureteral tearing, perforation, and infection. Cystoscopy, despite its invasiveness, remains superior in detecting small and superficial lesions. Ultrasound is also a diagnostic method for UC, with typical findings of hyperechoic calcifications within or around lesions and enhanced blood flow signals. Case 2’s penile ultrasound revealed a well-defined hypoechoic area with minimal blood flow. Urine cytology is useful for detecting high-grade bladder cancer and in situ carcinoma that may be challenging to diagnose under cystoscopy.^[9]^ In pathological examination, UC manifests with observed enlargement of cell nuclei accompanied by mild pleomorphism, loss of cell polarity, irregular morphology, and evident nucleoli. Additionally, fibrovascular nodules are observed.^[10]^ With the further development of technology, it has been reported that liquid based cytology (including circulating tumor cells, free deoxyribonucleic acid, free ribonucleic acid, extracellular vesicles, proteins and metabolites can be found in blood, urine or other body fluids) is a diagnostic method that can accurately diagnose the molecular profile of UC, which can supplement existing diagnostic techniques and benefit UC patients.^[11]^ Diffusion-weighted magnetic resonance imaging, as a new diagnostic technique, has been gradually applied to the management of upper urinary tract transitional cell carcinoma and bladder cancer, providing useful diagnostic information for the presence of malignant lesions in a noninvasive manner.^[12]^ Hence, clinicians should choose appropriate auxiliary diagnostic methods to promptly diagnose urinary tract epithelial carcinoma, preventing disease progression and the occurrence of urethral metastatic recurrence.

Currently, complete surgical resection is the optimal treatment for UC. As UC often extends beyond visible margins, thorough resection of the tumor margin is essential, and margin pathology examination confirms the presence of residual tumor tissue. Although all 3 cases underwent radical surgery, postoperative urethral metastasis occurred. The cause may be inadequate tumor resection, lack of adjuvant chemoradiotherapy in the abdominal and tumor bed areas. For patients with urethral metastasis, a comprehensive treatment plan involving total urethrectomy, chemotherapy, and immunotherapy is recommended for better prognosis, potentially achieving complete remission. According to the 2023 guidelines from the European Association of Urology, GC regimen is recommended as the first-line therapy for recurrent and metastatic patients.^[13]^ Cases 2 and 3 received GC chemotherapy after total urethrectomy, showing no local recurrence or metastasis in recent outpatient follow-ups, with good efficacy and satisfactory postoperative quality of life. In recent years, genomic research has identified numerous promising targets for UC, providing new opportunities for targeted therapies.^[14]^ These therapeutic targets include Atezolizumab (programmed cell death protein [PD-1] inhibitor), vofatamab (targeting FGFR), and various antibody-drug conjugates (ADCs).^[15]^ Recent studies have found that PD-1 + ADC combination therapy has shown promising efficacy in patients with UC who are CerbB-2 positive.^[16]^ In addition, PD-1 combined with ADC therapy has been found to significantly improve overall survival in patients with advanced CerbB-2 positive UC, and is a viable and safe bladder-preserving therapy, especially for patients with stage T2 MIBC who are not suitable for surgery and chemotherapy.^[17,18]^ For example, in the case of Patient 1, the immunohistochemistry staining results indicated CerbB-2 (2+), in view of this result, we consulted the relevant treatment guidelines and discussed from the aspects of patients’ economic ability，finally we utilized a combination adjuvant therapy comprising toripalimab (PD-1 inhibitors) 240 mg intravenous infusion and disitamab vedotin for injection (ADC drug) 120 mg showed no evidence of tumor recurrence or metastasis during regular follow-ups, and the patient’s quality of life remained satisfactory. In the case of Patient 3, postoperative pathology revealed negative CerbB-2 expression, leading to the decision not to pursue targeted therapy. Although the pathology examination for Patient 2 indicated CerbB-2 at 2+, due to considerations regarding the patient’s family’s financial situation and the refusal of the patient’s family members to accept immunotherapy, targeted therapy was not initiated in the treatment plan. In addition to these monoclonal antibodies for treating UC, other research has shown that atezolizumab has demonstrated improvements in postoperative metastasis, recurrence, and overall survival in patients with UC.^[19,20]^ The importance of postoperative follow-up for patients with advanced urinary tract epithelial cancer cannot be overstated. Postoperative follow-up is a crucial medical management activity, and regular follow-ups play a key role in assessing recovery, monitoring cancer recurrence and progression, providing psychological support, and delivering health education. This comprehensive approach contributes to caring for the patient’s physical and mental health, ultimately enhancing the overall effectiveness of treatment.

## 4. Conclusion

UC is prone to urethral metastasis and recurrence. Postoperative meticulous follow-up, including regular abdominal CT scans and cystoscopy, plays a crucial role in reducing the risk of recurrence and metastasis, as well as improving the prognosis and quality of life for patients. For patients with CerbB-2 positivity, PD-1 inhibitors + ADC combination therapy is also considered an effective adjuvant treatment option. In cases of postoperative urethral metastasis recurrence in UC, a comprehensive treatment strategy involving total urethrectomy combined with chemotherapy and immunotherapy proves to be an effective therapeutic approach.

## Author contributions

**Data curation:** Mengyao Li, Shouhua Pan, Keyuan Zhao.

**Formal analysis:** Mengyao Li.

**Funding acquisition:** Shouhua Pan.

**Visualization:** Shouhua Pan.

**Writing – original draft:** Jiajun Chen, Weihao Wang.

**Writing – review & editing:** Jiajun Chen.
